# Definition of apical descent in women with and without previous hysterectomy: A retrospective analysis

**DOI:** 10.1371/journal.pone.0213617

**Published:** 2019-03-15

**Authors:** Gerda Trutnovsky, Kristy P. Robledo, Ka Lai Shek, Hans Peter Dietz

**Affiliations:** 1 Department of Obstetrics and Gynaecology, Medical University of Graz, Graz, Austria; 2 Department of Obstetrics and Gynaecology, Nepean Clinical School, University of Sydney, Sydney, NSW, Australia; 3 NHMRC Clinical Trials Centre, University of Sydney, Sydney, NSW, Australia; 4 Department of Obstetrics and Gynaecology, Liverpool Clinical School, Western Sydney University, Liverpool, NSW, Australia; Weill Cornell Medical College, UNITED STATES

## Abstract

**Background:**

While normal pelvic organ support has been defined for women with intact uterus, this is not the case for post- hysterectomy vault descent. A recent systematic review found that definitions of apical prolapse are highly variable.

**Objectives:**

To investigate the relationship between prolapse symptoms and apical POP-Q measurements and establish cutoffs for ‘significant apical descent using receiver–operator characteristics (ROC) statistics.

**Study design:**

Retrospective analysis of patients seen at a tertiary urogynecological unit. Evaluation included a standardized interview and clinical assessment using the Pelvic Organ Prolapse Quantification (POP-Q) system. ROC curves were prepared for the relationship between prolapse symptoms and POP-Q measure “C”.

**Results:**

The records of 3010 women were available for analysis. Prolapse symptoms were reported by 52.3% (n = 1573), with a mean bother of 5.9 (SD 3.0, range 0–10). POP-Q point “C” was associated with symptoms of prolapse (p <0.0001) and prolapse bother (p <0.0001) on both univariate and multivariate analysis. ROC curves for women with and without uterus were similar, although the relationship between apical descent and symptoms of prolapse was stronger for women with uterus (AUC 0.728 versus 0.678). After controlling for multi-compartment prolapse, the models improved, resulting in AUCs of 0.782 and 0.720. For prediction of prolapse symptoms, cutoffs were set at C = -5 (sensitivity 0.73, specificity 0.67 with uterus in situ, sensitivity 0.59, specificity, 0.73 after hysterectomy).

**Conclusion:**

A cut- off for ‘significant central compartment descent’ of 5 cm above the hymen on Valsalva seems valid regardless of previous hysterectomy.

## Introduction

The prevalence of pelvic organ prolapse (POP) is increasing due to increasing life-expectancy and changing demographics [1, 2], and the lifetime risk for undergoing surgery for POP is now between 12% and 19% in Western countries [3, 4]. The degree and symptoms of POP vary considerably, from being asymptomatic to substantial negative impact on women´s physical, psychological, and social wellbeing [5–8]. Symptomatic prolapse is commonly associated with a feeling of a vaginal bulge, lump or a dragging pelvic sensation, but the relationship with prolapse determined on clinical examination is still widely discussed [8–12]. POP can occur at the anterior vaginal wall (cystocele), the posterior vaginal wall (rectocele) or at the apex (uterine or vault descent), and often involves more than one compartment. Although anterior prolapse is commonly the most pronounced, apical descent is considered important for POP symptoms and sexual function and needs to be adequately addressed [10, 12, 13]. However, a recent systematic review found that definitions of clinically significant apical prolapse for study inclusion and outcome assessment are highly variable. Currently there are no guidelines regarding the degree of apical support loss that should be considered ‘abnormal’, and further research is needed to develop evidence-based definitions for clinical practice and research [12].

The ICS POP Quantification (POP-Q) system defines the position of the most distal portion of the prolapse in each compartment in relation to the level of the hymen, and classifies prolapse into stages 0–4. In parous women anterior and/or posterior compartment prolapse stage 1 is common and mostly asymptomatic and suggested cutoffs for predicting prolapse symptoms range between 0.5 cm proximal and 0.5 cm distal to the hymen [6, 10, 14]. In contrast, stage 1 prolapse of the uterus seems to be highly significant and 5 cm above the hymen has been suggested as optimal cutoff for uterine prolapse [6]. However, currently there is little data on vault prolapse and its association with prolapse symptoms and bother. POP-Q point C refers to the cervix or vault after hysterectomy, but it is uncertain whether descent of Point C to a given level causes similar symptoms in women with and without uterus.

This study was undertaken to investigate the relationship between prolapse symptoms and apical ICS POP-Q measurement in women with and without previous hysterectomy.

The aim was to establish optimal cut- off levels for predicting symptoms and bother of prolapse and hereby define ‘normality’ using receiver–operating characteristics (ROC) curves.

## Materials and methods

This was a retrospective analysis of patients seen for assessment of lower urinary tract or pelvic floor disorders between March 2011 and December 2017 in a tertiary urogynaecological unit. Evaluation included a standardized interview, a clinical assessment using the ICS Pelvic Organ Prolapse Quantification (POP-Q) system [15] and 4 D translabial ultrasound, using GE 730 Expert system(GE Kretztechnik GmbH, Zipf, Austria). The comprehensive obstetric and urogynaecological history included details of previous deliveries, hysterectomy, prolapse and/or incontinence operations. The symptom of prolapse was ascertained by a standardized query of a “sensation of a lump or a bulge” and/ or a “dragging sensation in the vagina”. Subjective bother of POP was assessed by continuous Visual Analogue Scale (VAS) ranging from 0, “no bother at all” to 10, “worst imaginable bother” [7]. In patients reporting no prolapse symptoms the bother was assumed to be zero. For POP-Q measurements a wooden, disposable, calibrated ruler was used (PopStix; Endoventure, Auckland, New Zealand). To ensure accurate assessment, all Valsalva maneuvers were performed supine after voiding, at a minimum duration of 6 seconds [16]. Levator ani muscle avulsion was diagnosed using tomographic ultrasound imaging on volumes obtained during pelvic floor muscle contraction [17].

For statistical analysis, the POP-Q measure “C“, which relates to the position of the cervix or vault relative to the hymen, was regarded as the explanatory variable, with symptoms of prolapse (Y/N) being the main outcome variable. Logistic regression was used to test for univariate associations between prolapse symptoms and patient risk factors: age, BMI, parity, previous forceps delivery, avulsion and previous hysterectomy and previous prolapse/incontinence surgery. Receiver–operator characteristics (ROC) curves were prepared for symptoms of prolapse and C in women with and without previous hysterectomy, controlling for risk factors identified on univariate analysis. The areas under the ROC curve (AUC) were calculated as a measure of predictive performance, and cutoffs were determined. To control for multi-compartment prolapse, ROC statistics were repeated after excluding women with dominant prolapse in other compartments (defined as POP-Q ≥ 2 stage higher than uterine/ vault prolapse), leaving 2050 women for analyses. The relationship between VAS prolapse bother score and POP-Q measures was also assessed using univariate and multivariate linear regression analysis.

Statistical analysis was carried out using the software SAS V 9.3 (SAS Institute, Cary, NC, USA) and SPSS V 20 (SPSS, Chicago, IL, USA) and p<0.05 was considered statistically significant. Logistic regression analysis and ROC statistics were performed. The study was part of a retrospective study approved by the local human research ethics committee (Nepean Blue Mountains Local Health District 13–70). No consent was obtained, because the study was undertaken retrospectively and analyzed anonymously.

## Results

The records of 3010 women presenting during the study period were available for analysis. Women were predominantly of Caucasian origin, and from diverse socioeconomic backgrounds. Mean age was 56.9 (SD 13.5, range 17–89) years, with 64.4% (n = 1937) being postmenopausal, and the mean body mass index (BMI) was 29.1 (SD 6.4, range 14.7–67.8). Median parity was 2 (range 0–9) with 89.5% vaginally parous and 26.3% (n = 792) reporting at least one forceps delivery. Previous hysterectomy was reported by 947 women (31.5%), and 690 (22.9%) had had previous prolapse and/or incontinence surgery.

Prolapse symptoms were reported by 52.3% (n = 1573), with a mean bother of 5.9 (SD 3.0, range 0–10). On clinical examination the following POP-Q measures were recorded: “C”: mean -4.3 (SD 2.9, range -11 to +15); “Ba”: mean -0.7 (SD 1.7, range -3 to 8), “Bp”: mean -1.1 (SD 1.4, range -3 to 9). Levator muscle avulsion was diagnosed in 716 (23.8%), being unilateral in 457 (15.2%) and bilateral in 259 (8.6%).

**Fig 1:**
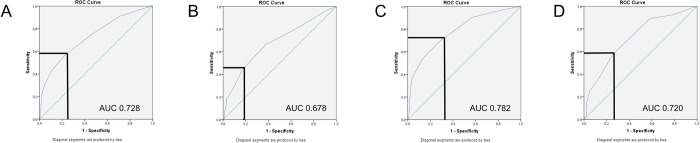
ROC curves for apical descent as a test for symptomatic prolapse. Bold lines define cutoff (C = -5). (A) women with uterus in situ, (B) women after hysterectomy. After exclusion of women with dominant prolapse in other compartments, similar results were obtained in women with uterus in situ (C) and after hysterectomy (D) (n = 2050).

On univariate analysis, apical descent, i.e. POP-Q point “C”, was highly significantly associated with symptoms of prolapse (p <0.0001) and ROC statistics resulted in an AUC of 0.711 (95% CI 0.693–0.729). ROC curves for women with and without uterus were similar, although the relationship between apical descent and symptoms of prolapse was stronger for women with uterus (AUC 0.728 versus 0.678). We determined cut- offs for ‘significant prolapse’ of C = -5 for women with uterus in situ (sensitivity 0.59, specificity 0.74) and after hysterectomy (sensitivity 0.45, specificity, 0.82), with a cut-off of -5.5 performing marginally better (sensitivity 0.67, specificity 0.61) after hysterectomy, see Fig 1.

Controlling for multi-compartment prolapse (n = 2050) further improved the models for “C” and prolapse symptoms, resulting in an ROC AUC of 0.782 and 0.720 for women with uterine and vault prolapse, respectively. For prediction of prolapse symptoms in this subgroup, cutoffs were set at C = -5 (sensitivity 0.73, specificity 0.67) and after hysterectomy (sensitivity 0.59, specificity, 0.73), see Fig 1. Controlling for BMI, parity, hysterectomy and avulsion in a multivariate logistic model, resulted in a slight improvement of ROC AUC to 0.743 (95% CI: 0.725–0.761) for the full dataset. Exclusion of women with previous pelvic floor surgery, i.e. anti-incontinence/ prolapse surgery and hysterectomy, did not alter model performance.

Linear regression analysis of VAS scores for prolapse bother showed highly significant relationships with POP-Q measures “C”, “Ba” and “Bp”, but also risk factors age, BMI, parity, avulsion, previous forceps delivery and previous hysterectomy (see Table 1). All POP-Q measures remained significant (<0.0001) after adjusting for these potential confounders on multivariate analysis.

**Table 1:** Linear regression analysis of factors predicting prolapse bother in 3010 women assessed for pelvic organ prolapse.

Model	Beta (95% CI)	P
C (per cm increase)	0.44 (0.40–0.48)	<0.0001
Ba (per cm increase)	0.88 (0.81–0.95)	<0.0001
Bp per cm increase	0.55 (0.46–0.64)	<0.0001
Age (per 1 year increase)	0.01 (0.01–0.02)	0.003
BMI (per 1 unit increase)	-0.03 (-0.05- -0.01)	0.003
Parity (0, 1 or > = 2)	0.88 (0.62–1.14)	<0.0001
Avulsion (no, unilateral, bilateral)	1.32 (1.11–1.52)	<0.0001
Previous forceps delivery	0.75 (0.46–1.05)	<0.0001
Previous hysterectomy	0.32 (0.04–0.59)	0.03

Beta gives the mean increase in VAS bother score (in cm on a scale of 0–10) for every unit increase in explanatory variable, or for the women with the condition vs those without it. Points Ba and Bp are the lowest points on the anterior and posterior vaginal walls, respectively. Point C represents the cervix or the vault. BMI, body mass index.

## Discussion

An evidence-based definition of clinically significant apical prolapse is important for both clinical care and research, and needs to be valid for women with and without uterus [8, 11]. In this large retrospective study in a symptomatic population presenting to a urogynecologic service, we correlated ICS POP-Q measures of uterine and vault descent with symptoms and bother of POP, using a consecutive dataset of over 3000 patients. In an attempt to optimize both sensitivity and specificity of a diagnosis of clinically significant apical prolapse, we applied ROC statistics while controlling for confounders, especially prolapse in other compartments. Our results demonstrate that a proposed cutoff of 5 cm above the hymen is not only valid for uterine prolapse but also for vault prolapse.

It is recognized that this cutoff is distinct higher than previously suggested cutoffs for prediction of POP symptoms using ROC statistics [6, 10, 18]. In the Pelvic Organ Support Study by Swift et al, which assessed over 1000 women in a general US gynecologic clinic population, POP was determined by a leading edge of the vaginal wall 0.5 cm above the hymen [10]. In a cross-sectional study of 296 women, vaginal descent 0.5 cm distal to the hymen was found to predict bulging/protrusion symptoms [14]. However, in both studies central descent was not separately assessed and POP symptoms were likely related to anterior or posterior descent.

Translabial ultrasound is another easily accessible, safe and inexpensive technique for specifying the degree of prolapse in each compartment, and cutoffs for clinically relevant prolapse have been established using ROC statistics [19, 20]. For prediction of prolapse symptoms due to uterine descent the optimal cutoff was defined as cervical descent to 15 mm above the symphysis pubis, which is 2.5 and 3 cm higher than cutoffs for significant cystocele (10 mm below) and rectocele (15 mm below the symphysis), respectively [19, 20]. Hence, imaging data strongly supports more cranial cut- offs for apical descent compared to anterior and posterior compartment descent.

In a recent systematic review the definitions of clinically significant apical prolapse for study inclusion and surgical success and failure were analyzed. In 35 randomized controlled trials the definitions were found to be either absent or highly variable, ranging from apical prolapse >-1cm to >+ 1 cm to > 50% of total vaginal length. The presence of prolapse symptoms as an inclusion or surgical success criteria was used in 46% and 29% of studies, only [12].

Our results highlight the need to consider compartments separately from each other and define specific cutoffs for apical and anterior/posterior descent. As far as the authors are aware, this is the first study to do so. ROC statistics for uterine descent and prolapse symptoms have been described using translabial ultrasound [19]. However, the vaginal vault is less visible on ultrasound than the cervix, and women after hysterectomy were excluded from the analysis. In our study we analyzed POP-Q data acquired using optimal valsalva maneuvers[16] and POP symptoms ascertained by a standardized query of a “sensation of a lump or a bulge” and/ or a “dragging sensation in the vagina”. POP related bother was assessed using VAS, which was shown to be a valid tool for determining subjective severity of POP in women [7]. However, no questionnaires on quality of life and/or sexual function were used, which may be considered a weakness.

The study is also limited by the retrospective design and the tertiary urogynaecologic unit setting. The patients were predominantly of Caucasian origin, and results may only pertain to similar populations. Interviews and clinical examination were not consistently blinded against each other, and this may have introduced measurement bias.

A considerable strength of the study is the large consecutive dataset of 3010 patients. This sample size allowed us to examine women with and without uterus separately, and excluding women with dominant prolapse in other compartments.

In conclusion, the previously proposed cut- off for ‘significant central compartment descent’ of 5 cm above the hymen on Valsalva seems valid regardless of previous hysterectomy. Prospective studies with standardized detailed questionnaires should help to confirm these findings.
